# Notes and News

**Published:** 1893-03-11

**Authors:** 


### Notes and News.

Collected and Communicated.

Mr. Passmore Edwards has again added to the list of his generous donations to his countrymen by the gift of a cottage hospital for eight beds to the town of Falmouth.

A meeting was held recently at Cannes, when it was decided to erect a small British hospital there. The Bishop of Gibraltar presided, and Sir Sydney Waterlow and Lord Ducie, and other influential persons were present. The hospital is to be for contagious and infectious diseases, and will especially meet the requirements of the new French law, which enforces isolation at the sufferer's expenses. It is hoped that the building will be erected by December next. The cost is estimated at £2,000, and one half of this sum was announced as subscribed at the meeting.

The monster baby of Western civilization, the World's Fair, is growing, and growing with such an apparent capacity for expansion in all directions, that where the limit is to be seems not very apparent. The great possibilities afforded by the site, caused a cordial welcome to be given to all would-be exhibitors in the early days of the scheme of arrangement. The amount of enterprise which such an opportunity for display has brought forth, is becoming alike embarrassing to the management and to the prospective visitor. It is very evident that to see even half the exhibits of interest a lengthy stay at Chicago will be necessary. Such a variety of exhibits are to be collected as perhaps have never come together before in the world's history. If we admire anything particularly nowadays we are quite surprised if we hear it is not going to Chicago. And yet the prohibited are perhaps nearly equal in number to the exhibited. One rejected show we think would certainly have attracted a great amount of attention. A cosmetic vendor applied to exhibit an old woman, half of whose face was to be smoothed out by his preparations, and the remainder with all its wrinkles was to be left untouched until the end of the Fair, when both sides of the face were to be made "beautiful for ever." Surely such rejected exhibitors will establish a World's Fair of their own.

Some time ago it was decided to build a hospital for the poor sick Jews outside the walls of Jerusalem, on the Jaffa Road. The site has been purchased and £5,000 subscribed towards the building fund. In order to proceed, a firman is necessary, and in procuring this a long and inexplicable delay has occurred. The question as to the delay has been brought forward before Parliament, and the matter thus brought into prominent notice, it is to be hoped that the building will soon be proceeded with.

The annual meeting of the Hospital Saturday Fund was held in the Mansion House on the 25th ult., the Lord Mayor presiding. The meeting was very fully attended, and the report read was of a most satisfactory character. The management show increasing energy, and the increase in the collections during the past year testify to the growing popularity of the fund. Mr. R. Acland, chairman of the Fund, stated that 7,000 firms had been visited, and a number of these would take up the work of collection in the future. Canon Erskine Clark advocated a system of weekly subscriptions in aid of the Metropolitan medical charities throughout the workshops, warehouses, and places of business. His suggestion was unanimously adopted.

The Duke of Westminster makes a special appeal, as President, for the Belgrave Hospital for Children. The institution does a great deal of work in the populous locality in which it is situated, and ever since its foundation extension after extension has been necessary, making the task of fitting income to expenditure exceptionally difficult. It is not surprising, therefore, to learn that the hospital is much in want of funds at the present time, the invested capital having been expended. The working classes testify to their appreciation of the care betowed on the little sufferers by large contributions, and this appreciation will doubtless also be shown in a substantial manner when the wants of the hospital are realised.

Whilst the Glasgow Royal Infirmary is to be condoled with on the serious deficit of £11,000 on the expenditure of the present year, the City is to be complimented on the public spirit which is being shown in the matter. If in the multitude of councillors there is indeed wisdom to be found, the way out of the difficulty will soon be clear. The Glasgow Press is opening its columns to the expression of all opinions, and there is no lack of suggestions from all sides. There are two striking facts brought out by the light brought to bear on the subject, and these are, firstly, that the infirmary opens its doors very largely to other than the inhabitants of Glasgow, and, secondly, that of the 700,000 citizens but 3,000 contribute £1 and upwards to the funds of the infirmary. Both these facts, now thoroughly grasped, open up a field for energetic work on the part of the management, and those interested in the infirmary, which affords help to so many, and is constantly increasing its sphere of usefulness in a manner which should gain the utmost recognition in return from grateful recipients of benefits direct and indirect. It may be added that the deficiency of £11,000 is due mainly to the special system of finance adopted by the managers, as a reference to the accounts, and especially to the sums invested, will show at once. The infirmary is, in fact, probably as prosperous as any hospital in Glasgow.

